# Appendicitis

**Published:** 1900-11-10

**Authors:** 


### APPENDICITIS.

There are various ways of classifying cases of appendicitis. Mr. Littlewood,[1] surgeon to the Leeds General Infirmary, divides them into (1) simple non-suppurative appendicitis, (2) suppurative appendicitis, (3) gangrenous appendicitis; while Mr. Mansell Moullin, in a paper read at the last meeting of the Harveian Society, also divides them into three groups, which differ somewhat in their lines of demarcation from those of Littlewood's classification, but agree with it in this, that in only the first form is it considered wise or even justifiable to leave the patient to medical treatment alone. He says that the first and by far the largest group includes mild attacks which subside of themselves or under the simplest treatment. The second group includes cases in which the absorption of the exudation is incomplete, in some becoming organised, in others breaking down into pus, and others in which a concretion is left behind. In all these perfect recovery is impossible without operation. The third and by far the smallest group includes those in which acute septic peritonitis breaks out, cases in which the only chance of recovery lies in immediate operation. Of course the difficulty is to decide in which of these classes a case is to be placed. Mr. Littlewood says that if there is no improvement in the patient's condition in forty-eight hours from the onset of the attack those who will be responsible for the operation should have a chance of seeing the case and of deciding whether or not an operation should be performed, and adds that while he has never regretted operating he has often regretted that operation had not been decided upon earlier. Mr. Mansell Moullin, however, gives a somewhat earlier time limit, saying that in all acute attacks it is absolutely essential to place the cases into one or other of the three groups above given within thirty-six hours of the onset of the symptoms, or it might be too late, and he urges that the danger is not in performing a simple exploratory operation, but in delaying to do so. All that is necessary, he says, is an incision an inch and a half in length, and the introduction of the finger, in order to ascertain exactly the condition of the appendix and of the peritoneum around it. The limit of thirty-six hours is, he says, an arbitrary one: all cannot wait so long, others can wait longer; but it is better that a simple and practically safe operation should be performed at once in all cases rather than that the case should be allowed to run its course, and those in which diffuse septic peritonitis occurs be allowed to die, in order to save the others from an operation which would have to be undergone later. As to general symptoms specially calling for operation he lays stress upon the pulse. If its rate at the end of thirty-six hours while the patient is lying in bed is over 100 a minute, or if in the course of the last few hours it has increased much in frequency, there is no doubt that the attack is a severe one, and that operation will be required. In reference to the importance of a quick pulse, especially when combined with a low temperature, Mr. Tubby[2] says: "In a case of declared appendicitis *the danger is extreme when the temperature is subnormal and the pulse over* 100. The moment you see such a case as this, you should be aware that you are dealing with a virulent case of appendicitis, a case accompanied by acute general peritonitis. There is no more definite danger signal than those two combined conditions of pulse and temperature. If you are well aware of this fact, you will have no excuse for delaying the calling in of surgical assistance. This rule is absolute and definite; an operation should be done at once." The virulent cases here referred to by Mr. Tubby are, of course, in a somewhat different category to those of which Mr. Mansell Moullin was speaking, but the rule remains as to the dominant importance of the pulse in this disease. This also has to be borne in mind, that although pain, tenderness, muscular resistance, sense of fulness in the right iliac region, vomiting, &c., may point strongly to the acuteness of an attack, the absence of no one such symptom in the presence of a rapid pulse can be taken as indicating that the attack is not of such severity as to require operation.

[1] Lancet, October 27. [2] Clinical Journal, Oct. 21st.

